# Lymphocytes in Cellular Therapy: Functional Regulation of CAR T Cells

**DOI:** 10.3389/fimmu.2018.03180

**Published:** 2019-01-18

**Authors:** Alka Dwivedi, Atharva Karulkar, Sarbari Ghosh, Afrin Rafiq, Rahul Purwar

**Affiliations:** Department of Biosciences and Bioengineering, Indian Institute of Technology Bombay, Mumbai, India

**Keywords:** chimeric antigen receptor, cancer immunotherapy, immunoregulation, anti-tumor efficacy, cytokines

## Abstract

Lymphocytes especially autologous T cells have been used for the treatment of numerous indications including cancers, autoimmune disorders and infectious diseases. Very recently, FDA approved Chimeric Antigen Receptor T cells (CAR T cells) therapy for relapse and refractory CD19+ B cell acute lymphoblastic leukemia (r/r B-ALL) and r/r diffuse large B cell lymphoma (r/r DLBCL) upon their remarkable success in multiple Phase I-II clinical trials. While CAR T cells are considered as major breakthrough in the field of cancer immunotherapy, the regulation of CAR T cells remains poorly understood. In this review we will discuss the strategies that regulate the CAR T cells efficacy and persistence with focus on roles of different structural component of CAR construct. Different domains of CAR construct, for example, antigen binding domain, hinge, transmembrane, and signaling domain as well as immune-regulatory cytokines have significant impact on CAR T cell efficacy. Finally, this review will highlight the strategies that will promote CAR T cells efficacy and will reduce the toxicity.

## Introduction

Chimeric antigen receptor T cells (CAR T cells) have achieved remarkable success in the field of cancer tumor immunotherapy since last decade (1). Promising clinical outcomes were observed in case of hematological malignancies leading to FDA-approval of Kymriah™ and Yescarta™ for r/r B-ALL in pediatric and young adults and adult patients with large B cell lymphoma, respectively. This has led to a paradigm shift in the field of cancer immunotherapy especially in treatment of some hematological cancers across the globe. The enormous success of CAR T cells in hematological malignancies is attributed to numerous factors, most important being the choice of CD19 expression on all B cells (2, 3). Other factors are easy sampling of the tumor and trouble free homing of the T cells to hematologic organs such as blood, bone marrow and the lymph nodes (4). On the contrary, efficacy of CAR T cells targeting solid tumors is still in its infancy due to multiple challenges such as lack of unique tumor associated antigen (TAAs), inefficient T cell homing to the tumor bed and due to immunosuppressive tumor microenvironment. These factors lead to limited persistence and sub-optimal efficacy of the CAR T cells in solid tumor settings (5).

**Figure 1:**
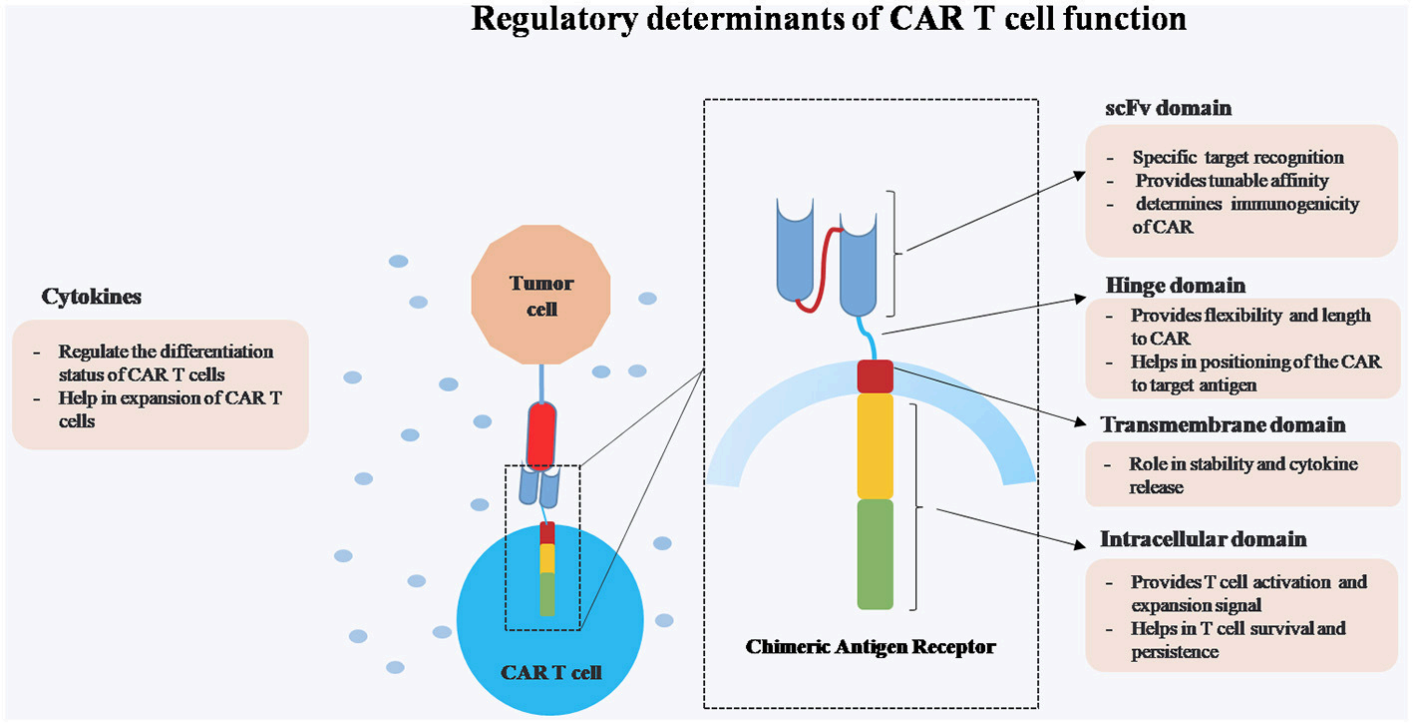
Schematic representation of different regulatory components of Chimeric Antigen Receptor (CAR) T cells.

CARs consist of four main domains, namely ectodomain for specific target antigen recognition and endodomain that provides costimulatory and activation signals. These two domains are connected by hinge and transmembrane domain (Figure 1). The major advantages of CAR T cells over other cell based therapies are (1) killing of tumor targets in a MHC independent manner and thereby overcoming certain tumor escape mechanisms such as MHC-I down regulation and faulty antigen processing, (2) engineering of multiple anti-tumor immuno-modulators, and (3) targeting wide array of antigens (protein, carbohydrate, and glycolipid).

While CAR T cells are effective in achieving long-term remission in certain types of malignancies, the major challenge remains in controlling CAR T cells in case of dysregulated activation. Hence, our major focus in this review is to elucidate the structure-function relationship of different components of CAR construct on CAR T cells persistence and efficacy.

### Impact of ScFv Ectodomain on CAR T Cell Functions

The scFv ectodomain, which plays important role in the efficacy and safety of CAR T cells, is a smallest synthetic functional module containing variable heavy (VH) as well as variable light (VL) chain portion of an antibody linked with a long flexible linker. Most commonly used linker in several CAR construct is (Gly4Ser)_3_. Glycine residues provide flexibility and serine residues provide solubility. This renders a properly folded scFv thereby maintaining antigen-binding capability of the parental IgG. Differences in orientation of the VH, Linker and VL may affect the scFv's affinity and specificity (6). scFvs are critical for antigen specific CAR T cell activation. Non-specific or cross-reactive CARs can augment the off-target toxicity and inefficient CAR T cell activation. Hence, considerable efforts are needed for scFv designing and its characterization.

Two approaches especially animal immunization and surface display methods have been widely used for designing and screening of scFv. Although the most effective method is immunization, in case of well-conserved antigen there has been reports of generation of neutralizing antibodies due to cross reactivity (7).

There are various antibody display technologies such as phage display, cell display, ribosomal display, mRNA display and DNA display, out of which, phage display is one of the commonly used screening method (8–10). Display methodologies such as phage display overcomes the limitation of immune tolerance since they are based on *in vitro* selection from naïve or immune libraries (11). Yeast surface display emerged as an alternative technology to phage display, generating 10^8^−10^9^ library members. These antibodies have better affinity and specificity profiles through combination of library screening by flowcytometry and affinity maturation by *in vitro* codon variation or mating mediated chain shuffling (12, 13). In recent years high throughput eukaryotic cell display technologies have been successfully utilized. The advantage of this technology is real time analysis and characterization of library along with machineries for proper folding before being displayed on the surface of the cell. High throughput display technologies creates antibody libraries from which antibody fragments or domains can be selected for better effector function, tissue penetration and pharmacokinetics (14). Therefore, in order to cater the screening of antigen binding of scFv domains in CAR, either of the above methods have been utilized and have a significant role in deciding the CAR T cell efficacy.

The four important characteristics of scFv are immunogenicity, affinity, specificity, and its binding epitope. The monoclonal antibodies (mAbs) obtained from murine hybridomas were found to be immunonogenic in humans which resulted in low efficacy and immediate elimination from circulation (15, 16). They also showed systemic inflammatory responses resulting in serious physiological complications. Hence humanization of scFv can help to enhance safety and therapeutic potential of a CAR. Anti-folate receptor α (FRα) CAR T cells were developed against metastatic ovarian cancer using MOv18scFv which is a murine mAb for FRα. But, the CAR T cells showed poor persistence and anti-tumor efficacy (15, 17). In another study involving mesothelin-targeted CAR T cells containing SS1 (murine scFv), anaphylactic shock was observed in a patient. This was probably promoted by IgE antibodies specific for murine scFvs. This further indicates potential immunogenicity of murine scFv containing CARs (16). These CARs showed less *in vivo* persistence along with poor anti-tumor efficacy. Less immunogenicity was observed due to humanization resulting in enhanced persistence and safety of CAR T cells. A low affinity but highly specific CAR for epidermal growth factor receptor variant III (EGFRvIII) was humanized and included in the second-generation CAR T cells containing EGFRvIII scFv, 4-1BB and CD3ζ domains. Patients infused with this CAR showed minimum off-target toxicity and decreased cytokine release syndrome (18). The above humanized CARs showed better persistence and functionality but they still pose a risk of off-tumor toxicity owing to the 5% residual mouse sequences. This leads to the necessity of developing fully humanized scFvs, either from phage display or transgenic mouse models. In this connection, M28z CAR, consisting of m912 scFv (fully human anti-mesothelin mAb) was generated to resolve the immunogenicity issue which resulted in long term complete remission as reported in *in vivo* tumor models (19). Few other humanized CARs such as anti-FRα CAR for ovarian cancer and anti-CD22 CAR derived from m971 are in clinical trials (2, 3, 20, 21). With these advantages of using humanized scFv derived CARs, a case report of anti-HER2 CAR T cells containing scFv from trastuzumab (humanized mAb-herceptin) showed exceptional fatality with dosage of 1 × 10^10^ cells/infusion (22). In contrast to this, the patients receiving a low dose (1 × 10^8^ cells/m^2^) of anti-HER2 CAR T cells derived from murine clone FRP5 showed increased tolerance along with minimum toxicity (23). In response to this observation, the change in epitope binding affinity and avidity might have an impact on the efficacy and toxicity of the anti-HER2 CAR T cells. The epitope of HER-2 recognition is distinct for trastuzumab (derived from 4D5 clone) in comparison to murine FRP5 clone. Other factors which might have role in reduced toxicity are the T cells dosage, lack of lymphodepletion regime and less persistence of murine FRP5 anti-HER-2 CAR over trastuzumab containing CAR T cells. However, due to involvement of multiple factors in treatment, it remains unclear to identify the exact reason of the exceptional fatality using humanized scFv. Tumor associated antigens (TAAs) are the prominent targets for immunotherapy that are highly expressed on tumor tissue and also expressed at lower level in healthy tissues. This leads to unwanted recognition and sometimes life threatening toxicity. Therefore, the scFv selection is crucial while designing CARs in order to discriminate between tumor cells and normal tissues. To avoid these complications, the interactions between CAR scFvs and target antigens should be carefully understood and a cross reactivity study should be performed before finalization of scFv. One approach is to increase the scFv affinity toward target antigens. For example, CARs containing high affinity scFvs for receptor tyrosine kinase like orphan receptor I (ROR1) and folate receptor β (FRβ) have shown superior effector functions than CARs with low affinity scFvs (24, 25).

In addition, a fine balance between specificity and affinity for the target antigen also plays a determining role in CAR T cell function. Affinity tuned CARs having more specificity and less affinity showed high therapeutic index (26). Further, epitope positioning also determines the efficiency of CAR T cell activation. For example, scFv recognizing a CD22 epitope, lying proximal to B cell plasma membrane showed enhanced anti-tumor function as compared to one recognizing membrane-distal epitope (27). Therefore, the approach of incorporating a flexible linker in the CAR construct can modulate the antigen binding.

Upon the long term follow-up of the r/r B-ALL patients, it was observed that the tumors have acquired resistance to anti-CD19 CAR T cells (28–30). Possible reasons for the resistance toward anti-CD19 CAR T cell could be: (1) loss of target antigen**, (**2) exhaustion and lesser persistence of anti-CD19 CAR T cells (3) immunosuppressive tumor microenvironment (31, 32). To overcome the loss of antigen target due to mono-antigen specific CARs, bi-specific CARs have been designed to recognize two antigens in a true Boolean OR-gate fashion (i.e., either of the two antigens binding should be sufficient to trigger robust T-cell output). The studies have shown that in a high-disease burden setting, the bispecific CD19-CD20 CARs CAR proved both effective and less toxic than single CARs in pre-clinical settings(33, 34) Similarly, bispecific CAR has been designed to target both human epidermal growth receptor 2 (HER2) and IL13Rα2 and it showed enhanced potency and anti-tumor activity *in vivo* compared to two separate CARs (35). There are multiple on-going clinical trials in children and adults using bispecific CAR T cells (NCT03241940, NCT03233854, and NCT03448393). As reported in Phase 1 trial of anti-CD19/CD22 CAR T cells, anti-leukemic activity was observed with complete remission in 5/5 patients with CD19 dim/neg B-ALL for a duration of 6 months (20). Long term follow-up of these clinical trials will give the insights on superiority of bispecific CAR strategies over current monospecific CAR T cells.

Hence, these four aspects of scFv (immunogenicity, specificity, affinity, target positioning) are vital in determining the safety and functional efficacy of CAR T cells.

### Regulation by Hinge Region

Hinge region connects the ectodomain and the transmembrane domain of a CAR. Amino acid fragments from CD8α, IgG1, and IgG4 are the most common hinge used in majority of the CARs. Hinge region can affect the CAR function by providing flexibility and length to a CAR. Function of hinge region may vary depending on the targeted antigen. For example, hinge-less CARs against different antigens like CD19, carcinoembryonic antigen (CEA), neural cell adhesion molecule (NCAM) and 5T4 showed different effector functions compared to CARs having CH2CH3 hinge adapted from IgG1. CAR T cells against CD19 and 5T4 showed enhanced functional efficacy after adding CH2CH3 hinge in contrast to CEA and NCAM CAR T cells (36). As described in scFv section accessibility of a target antigen can modulate the efficacy of a CAR provided by a flexible linker as well as hinge region. Investigators have seen that in case of anti-CD22 CAR having a spacer derived from IgG1 Fc receptor impacts the positions of the targeted antigen with respect to cell surface and leads to increased efficacy (27). Moreover, length of hinge region is also critical for CAR T cells efficacy, shorter hinge region showed enhanced antitumor efficacy against ROR1 antigen compared with longer hinge (37). Very recently mesothelin CAR containing IgG4 hinge have shown higher efficacy and CAR T cell proliferation than the CAR without hinge region by bringing the mesothelin antigen proximal to membrane and thereby reducing the steric inhibitory effects between scFv and its target epitope (38). Recently investigators have found that replacing IgG1 hinge to IgG2 enhanced the efficacy further and reduced off target effect of a CAR against an antigen prostate stem cell antigen (PSCA) which is overexpressed on many solid tumors (39). These findings suggest that hinge region provides the flexibility to overcome the steric hindrances as well as reduces the distance between CAR scFv and antigen. Further studies are ongoing to understand the effect of hinge domain on flexibility, distance and elimination of the off target effects in relation to CARs.

### Roles of Transmembrane Domain of CAR Construct in CAR T Cell Function

Transmembrane region is a linker between hinge region and endodomain of a CAR. Type I proteins such as CD3ζ, CD28, and CD8α have been used as transmembrane domains in CAR constructs. Earlier it was thought that transmembrane domain does not have much impact on CAR T cell efficacy except anchoring CAR molecule to the membrane. But recent investigations have shown that transmembrane domain impacts the efficacy as well as stability of CARs (40). For example, in first generation CARs, CD3ζ, containing transmembrane domain dimerizes with endogenous TCR and enhances the CAR T cell function as compared to the mutated CD3ζ transmembrane domain (41). In addition to this Savoldo et al. have shown that CARs containing CD3ζ transmembrane domain are less stable on the cell surface as compared to the CD28 transmembrane domain in second generation CARs (42). According to Guedan et al. transmembrane domains helps in persistence as well as enhance antitumor efficacy in third generation ICOS based CAR. It was observed that the ICOS TM domain is required for the optimal *in vivo* phenotype of third generation ICOS based CAR T cells. However, ICOS transmembrane domain did not show any effect on CAR cell-surface expression or tonic signaling (43). Very recently investigators have demonstrated critical role of hinge and transmembrane domain in context of release of effector cytokines which is one of the crucial challenges of CAR T cell therapy known as cytokine release syndrome (CRS) that involves release of excess amounts of cytokines causing toxicity. CAR T cells having CD8α hinge and transmembrane domain release less IFN-γ and TNF-α as compared to those having CD28 hinge and transmembrane domain with no significant differences in CAR T cell efficacy and proliferation (44). Therefore, designing of a robust CAR should involve the best combination of hinge and transmembrane domain to regulate the functionality of CAR T cells.

### Regulation by Intracellular Endodomains

Different generations of CARs vary in their respective intracellular/co-stimulatory signaling domains. While first generation CARs contain only CD3ζ intracellular domain, the second and third generation CARs contain CD3ζ intracellular domain along with either single co-stimulatory domain or two co-stimulatory domains like CD28 and 4-1BB (4). Upon antigen binding phosphorylation cascade of immunoreceptor tyrosine-based activtion motif ITAMs present in CD3ζ intracellular domain is initiated leading to activation and priming of CAR T cells (45). However, second generation CAR modification over the first generation CARs by inserting the CD28 co-stimulatory domain demonstrated increased expansion and persistence of CAR T cells as compared to first generation counterparts (42).

On the contrary, 4-1BB (CD137), another co-stimulatory receptor is responsible for enhanced T cell survival. In a clinical study, the second generation CD19 CAR (CTL019) containing anti-CD19 scFv, CD3ζ domain along with 4-1BB co-stimulatory domain revealed robust expansion and long persistence of CTL019 cells along with sustained remissions in patients with relapsed/refractory chronic lymphocytic leukemia CLL (46). Another study using umbilical cord blood T cells (UCB T cells) revealed that UCB-19BBz and UCB-1928BBz CAR T cells were more cytotoxic toward CD19+ leukemic cell lines as compared to UCB-19z and UCB-1928z CAR T cells (47). Few studies compared the utility of CD28 and 4-1BB in various CAR constructs and data revealed the almost similar early response rates in ALL patients when treated with either CD28 or 4-1BB CAR (48, 49). However, in case of CLL, the 4-1BB CARs exhibited superior efficacy than the CD28 CARs, probably due to increased persistence of 4-1BB (CD137) CAR T cells and exhaustion of CD28 CAR T cells driven by CD28 endodomain signaling (46, 50, 51). The study done by Brentjens et al. in 8 CLL patients showed that infusion of 19-28z T cells resulted in complete reduction in lymphadenopathy in 1/8 patients (12.5%), progressive stable disease in 3/8 patients and no objective response in 4 patients. However, according to Porter et al., overall response rate in CLL patients when treated with CTL019 (CD19 scFv+ 4-1BB costimulatory domain) was found to be 8/14 (57%), where 4 patients showed complete remissions (CR) and 4 showed partial remissions (PR). The CAR T cells persisted and remained functional for more than 4 years in case of two patients who achieved CR and showed no relapse symptoms.

In another study, 4-1BB CARs (GD2-BBz CAR T cells) have been demonstrated to ameliorate CAR T cell exhaustion by decreasing the expression of exhaustion related molecules and by upregulation of three critical pathways such as hypoxia inducible signaling, cellular metabolism and negative apoptosis regulation (51). However, it remains poorly understood how these pathways contribute in ameliorating CAR T cell exhaustion in 4-1BB CARs.

In addition, CAR signaling impact metabolic reprogramming in T cells by modulating bioenergetics and mitochondrial biogenesis. The CD28z CAR T signaling facilitates differentiation of T_EM_ and increased aerobic glycolysis in T cells. On the contrary, 4-1BBz CAR T cells display differentiation to T_CM_ cells along with increased mitochondrial biogenesis and oxidative metabolism (52). The high rate of mitochondrial respiratory capacity as observed in case of 4-1BBz CAR T cells promotes CD8+ T cell memory differentiation and the oxidative phosphorylation acts a major source of energy thereby supporting increased CAR T cell proliferation (53). Therefore, in circumstances where long term CAR T cell persistence can cause severe off tumor toxicity, short-lived CARs can be designed by incorporating CD28 co-stimulatory domains.

Although many studies demonstrated that 4-1BB CAR is safe with increased persistence, in case of CD5+ tumor targeting, CD5-4-1BB CAR showed reduced efficacy compared with CD5-CD28z CAR due to enhanced T cell fratricide. To understand the mechanism of these observations, it has been described that the tumor necrosis factor receptor associated factor (TRAF) signaling induced by 4-1BB co-stimulatory domains upregulates expression of intracellular adhesion molecule 1 (ICAM-1) (usually not expressed on T cells) which in turn stabilizes the immunological synapse between CD5 CAR T cells (54). In order to circumvent this CAR (either 4-1BB or others) induced toxicity, a regulated CAR expression system may be developed.

While majority of available CARs either used 4-1BB or CD28 CARs, another CD4-related co-stimulatory receptor OX40 (CD134) emerged as a prominent approach in CAR signaling. Investigators have shown that a third generation CAR CD28-z-OX40 helped CCR7 (-) T cells avoid apoptosis and show potent anti-tumor functional efficacy (55, 56). Another report by the same group revealed that in comparison to a CD28-z CAR, the 3rd generation CD28-z-OX40 CAR decreased secretion of repressive cytokine like IL-10 without altering secretion of pro-inflammatory cytokines, T cell proliferation and cytotoxic potential. OX40 signaling further repressed Treg mediated IL10 secretion (56). This aspect of OX40 signaling can be harnessed in controlling Treg to effector T cell ratios in adoptive immunotherapy.

The ICOS expressing CARs induce ICOS signaling in the T cells and thereby increase the expression of IL-17A, IL-17F, and IL-22 (57). ICOS mediated co-stimulatory CAR signaling established Th17 characteristics such as increased expression of RORC, CD161, IL-1R1, and NCS-1. ICOS signaling also fostered Th17/Th1 polarization by enhancing IFN-γ and Tbet expression. *In vivo* animal studies further revealed enhanced persistence and anti-tumor responses of CD4+ CAR T cells in case of ICOS based CARs in contrast to the CD28 or 4-1BB containing CARs (43, 57). The extended persistence of ICOS-based CARs can be translated to non-lymphoid tumors where CAR T cell persistence is poorly understood. However, effect of ICOS based CARs is yet to be tested clinically in patients.

Hence, the co-stimulatory domains play a key regulatory role in determining the anti-tumor efficacy, functionality and persistence of CAR T cells both *in vitro* and *in vivo*. A thorough understanding of the signaling cascades associated with various co-stimulatory molecules will help in designing more effective CAR with better clinical implications.

### Regulating CAR T Cell Efficacy With Homeostatic Cytokines and Other Genes

Efficient T cell activation requires three signals, T cell receptor (TCR) signaling (Signal 1), and activation by co-stimulatory molecules (Signal 2) and immune-stimulatory cytokines (Signal 3). So far majority of the CARs designed and discussed possess signal 1 and signal 2, however, signal 3 generally provided by homeostatic cytokines is absent in the conventional CAR T cells and also less abundant in the tumor microenvironment (15) Therefore, next generation of CAR T cells would require an additional cytokine signaling to satisfy the need of signal 3 for optimal CAR T cell activation (58). The major cytokines involved in T cell activation belong to γc class like IL-2, IL-7, IL-15, IL-21, and IL-9. These cytokines control T cell survival and proliferation, which ultimately has significant roles in CAR T cell persistence and efficacy (59). These cytokines are currently employed in *ex vivo* expansion of CAR T cells prior to therapy in combinations or alone (60).

Starting with the well-studied cytokine in T cell activation and regulation, IL-2 has ranked first upon receiving a US FDA approval for employment in immunotherapy of melanoma in 1998 (61). However, along with induction of potent anti-tumor T cells, IL-2 is also known to cause Activation Induced Cell Death (AICD) and differentiation into immunosuppressive regulatory T cells (62). Hence, low dose IL-2 in CAR T cell therapy has been suggested for achieving better anti-tumor responses and to overcome the immunosuppressive effects of IL-2 (63).

Another approach to lessen the immunosuppressive effect of IL-2 on T lymphocyte is to make way for other cytokines like IL-7 and IL-15 in adoptive immunotherapy. With the discovery of T stem cell like memory cells (T_scm_) it has been described that IL-7 and IL-15 are known to generate and maintain less differentiated T_SCM_ population in the T lymphocyte pool thereby improving the antitumor responses significantly (64–66). In the absence of these regulatory cytokines the differentiation status of CAR T cells is skewed toward terminal differentiation thereby reducing functional potency of the CAR T cells. Owing to this fact, incorporation of IL-7 and IL-15 in the *in vivo* expansion of CAR T cells has been a new addition in the adoptive immunotherapy field (60).

Attempts have been made for administration of cytokines like IL-2, IL-7, IL-15, and IL-12 in clinical trials against various malignancies. However, the anti-tumor responses are greatly masked by toxicities generated due to the cytokine administration alone or along with CAR T cells (67–70). The toxic effects of recombinant cytokines are due to higher doses of i.v. administration in patients leading to off target toxicity. Therefore, attempts are been made to engineer CAR T cells to secrete or express these cytokines on the T cell surface giving rise to next generation of CAR T cells. A variant of IL-7R has been engineered to be expressed along with CAR T cells against solid tumors and has shown a good response in improving the anti-tumor efficacy *in vivo* models (71). CAR T cells secreting IL-15 have been tested in hematological malignancies along with anti-CD19 CAR expression. The IL-15 expression has shown to regulate differentiation of these modified CAR T cells by inhibiting apoptosis and showing reduced Treg induction unlike IL-2 (72). Along with the secretory form, the membrane bound expression of IL-15 has also shown to be a good strategy in achieving less differentiated CAR T cells and in generating potent anti-tumor responses in leukemia models (73).

In addition to this, IL-12 armored CAR T cells has shown to eliminate the need of pre-treatment regime before the onset of CAR T therapy in a preclinical setting. IL-12 armored CAR T cells also possess an intrinsic resistance to regulatory T cells mediated inhibition (74). However, another group of researchers conducting clinical trials with engineered TILs to secrete IL-12 showed a highly toxic response owing to the uncontrolled cytokine release (75). In addition, a report suggested leukemic transformation and clonal expansion of CD8+ T cells transduced to express IL-15 gene. This further helped in realizing the need for control of signal 3 generated by immune regulatory cytokines (76).

In order to regulate the adverse effects mediated by cytokine secretion as well as to regulate toxicities due to CAR T cells as observed in clinical trials, the scientists are designing strategies to tune these cells in a ligand dependent manner (77). One such “safety-switch” is the inducible caspase 9 molecule integrated with CAR T cells which can be controlled by an external ligand AP1903. Administration of the ligand induces caspase 9 mediated apoptotic pathway thereby clearing the CAR T cells from body and reducing the off-target cytotoxicity. These switches have been combined with cytokines IL-7 and IL-15 in the preclinical studies to provide a hope for managing toxicities due to the uncontrolled cytokine secretion (71, 78).

With such newer modifications in the CAR T cell designs, the focus of the field is gradually shifting toward regulation of actions of CAR T cells with presence of tunable signals and use of inhibitory receptors. Very recently, a new generation of CAR T cells, the SUPRA CAR T cells is been demonstrated (79). SUPRA stands for Split, Universal and Programmable CAR T cells, which can respond to multiple antigens and can be inducible controlled to manage T cell activation mediated toxicity. Other such CAR T cells such as iCARs (inhibitory CARs) are analyzed in preclinical studies wherein use of CTLA-4 and PD-1 based self-regulating inhibitory receptors have been employed to control off-target toxicities (80).

The current advances in the employment of cytokines and receptors thereof in regulating the CAR cell response is more on the preclinical front. The clinical outputs of these engineered CAR T cells along with newly designed switches would provide a useful insight into planning strategies for better regulation of CAR T cells in immunotherapy. Until then, the management of signal 3 for the regulation of CAR T cells stills remains elusive and opens a new field of research in adoptive immunotherapy.

## Conclusion and Future Prospective

Chimeric antigen receptors emerged as new genre of drugs with huge therapeutic potential for hematological malignancies as well as solid tumors. Different components of a CAR strongly impact the anti-tumor efficacy, potency and safety of the genetically engineered CAR T cells after infusion into the patients. This review highlights the importance of scFv designing in determining flexibility, affinity and specificity of the CAR along with strategies to minimize the off target toxicity. The regulatory role of hinge domain and transmembrane domain in providing proper positioning of the CAR, imparting stability and reducing cytokine release toxicity has also been discussed. Moreover, this review conveys the key role of intracellular domain in relation to long term survival and persistence of CAR T cells along with the regulatory aspects of various cytokines like IL-2, IL-15, IL-7, and IL-21. Fine tuning of the crucial components and parameters as discussed in this review will pave the way for developing more safe and efficacious CAR T cells for a wide array of malignancies.

Despite the successful achievements of CAR T cell therapy in case of hematological malignancies it displays to serious side effects such as cytokine release syndrome (CRS) that causes systemic inflammatory responses, multi-organ failure and death as well as neuro-toxic effects like aphasia, hallucinations and seizures (67). Another major concern is the “on target off-tumor” toxicity. The efficacy and safety of CAR T cells is monitored in preclinical animal models prior to its clinical testing, which includes syngeneic, human xenograft, immunocompetent transgenic, and humanized transgenic mice. These pre-clinical models neither reflect the obstacles in clinical efficacy nor do they predict potentially life-threatening safety concerns and hence lack to display the complications which might arise in the clinical setting. Moreover, the lack of host immune system does not allow testing of the tumor microenvironment, the tumor's metastatic potential, or the host response to CAR T cells. Each murine model has its own advantages and shortcomings which highlights the absence of a single competent model that can evaluate CAR T cell efficacy as well as toxicity issues. Recently, many primate models such as macaque or dog are also being studied in relation to CAR T side effects (81, 82). Hence, the evolution and refinement of preclinical models will lead to improved prediction of CAR T safety and efficacy in the clinic.

With all the evidences available with varied designs of the CAR in the field, so far it remains elusive to understand the use of correct combination of these domains which regulate the CAR T cell efficacy. In order to generate a predictive model to understand the efficacy and toxicity of the designed CAR T cells in *in vitro* experiments, arises the need to design computational biology tools. The development of computational approaches with a combination of clinical outcomes from the currently developed CAR T cells, may have a major impact in the designing superior CAR T cells with minimal toxicity and improved efficacy in future.

## Author Contributions

All authors listed have made a substantial, direct and intellectual contribution to the work, and approved it for publication.

### Conflict of Interest Statement

The authors declare that the research was conducted in the absence of any commercial or financial relationships that could be construed as a potential conflict of interest.
