# Corrigendum: Lymphocytes in Cellular Therapy: Functional Regulation of CAR T Cells

**DOI:** 10.3389/fimmu.2019.00401

**Published:** 2019-03-08

**Authors:** Alka Dwivedi, Atharva Karulkar, Sarbari Ghosh, Afrin Rafiq, Rahul Purwar

**Affiliations:** Department of Biosciences and Bioengineering, Indian Institute of Technology Bombay, Mumbai, India

**Keywords:** chimeric antigen receptor, cancer immunotherapy, immunoregulation, anti-tumor efficacy, cytokines

In the original article, author names were incorrectly published with the first and last names reversed. The correct authors names are: “Alka Dwivedi,” “Atharva Karulkar,” “Sarbari Ghosh,” “Afrin Rafiq,” and “Rahul Purwar.”

The authors apologize for this error and state that this does not change the scientific conclusions of the article in any way. The original article has been updated.

